# Bis[(18-crown-6-κ^6^ *O*)sodium] (18-crown-6-1κ^6^ *O*)-μ-thiocyanato-1:2κ^2^ *S*:*N*-pentathio­cyanato-2κ^5^ *N*-indate(III)sodium 1,2-dichloro­ethane sesquisolvate

**DOI:** 10.1107/S1600536809039282

**Published:** 2009-10-07

**Authors:** Lingqian Kong

**Affiliations:** a Dongchang College, Liaocheng University, Liaocheng 250059, People’s Republic of China

## Abstract

The title complex, [Na(C_12_H_24_O_6_)]_2_[InNa(NCS)_6_(C_12_H_24_O_6_)]·1.5C_2_H_4_Cl_2_, has been synthesized by the reaction of 18-crown-6 with InCl_3_ and NaSCN. The In atom has a six-coordinate octa­hedral environment, being bonded to the N atoms of six thio­cyanate groups. The bond lengths and angles show normal values. The crystal packing exhibits no significantly short inter­molecular contacts.

## Related literature

For background to crown ethers and their metal cations, see: Desai *et al.* (2001 ▶). For related structures, see: Mullica *et al.* (1999 ▶); Li & Dou (2003 ▶); Han *et al.* (1987 ▶, 1989 ▶); Noth & Warchhold (2004 ▶).
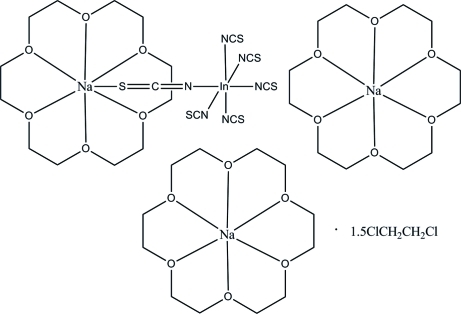


## Experimental

### 

#### Crystal data

[Na(C_12_H_24_O_6_)]_2_[InNa(NCS)_6_(C_12_H_24_O_6_)]·1.5C_2_H_4_Cl_2_
*M* *_r_* = 1473.63
Monoclinic, 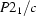

*a* = 13.7304 (15) Å
*b* = 22.144 (2) Å
*c* = 23.469 (3) Å
β = 90.541 (2)°
*V* = 7135.3 (13) Å^3^
*Z* = 4
Mo *K*α radiation
μ = 0.70 mm^−1^
*T* = 298 K
0.29 × 0.27 × 0.25 mm

#### Data collection

Bruker SMART CCD area-detector diffractometer
Absorption correction: multi-scan (*SADABS*; Sheldrick, 1996 ▶) *T*_min_ = 0.823, *T*_max_ = 0.845
37284 measured reflections
12551 independent reflections
6533 reflections with *I* > 2σ(*I*)
*R*_int_ = 0.051

#### Refinement

*R*[*F* ^2^ > 2σ(*F* ^2^)] = 0.071
*wR*(*F* ^2^) = 0.236
*S* = 1.02
12551 reflections
739 parameters
1314 restraints
H-atom parameters constrained
Δρ_max_ = 1.30 e Å^−3^
Δρ_min_ = −1.09 e Å^−3^

### 

Data collection: *SMART* (Siemens, 1996 ▶); cell refinement: *SAINT* (Siemens, 1996 ▶); data reduction: *SAINT*; program(s) used to solve structure: *SHELXS97* (Sheldrick, 2008 ▶); program(s) used to refine structure: *SHELXL97* (Sheldrick, 2008 ▶); molecular graphics: *SHELXTL* (Sheldrick, 2008 ▶); software used to prepare material for publication: *SHELXTL*.

## Supplementary Material

Crystal structure: contains datablocks I, global. DOI: 10.1107/S1600536809039282/ds2003sup1.cif

Structure factors: contains datablocks I. DOI: 10.1107/S1600536809039282/ds2003Isup2.hkl

Additional supplementary materials:  crystallographic information; 3D view; checkCIF report

## Figures and Tables

**Fig. 1.**
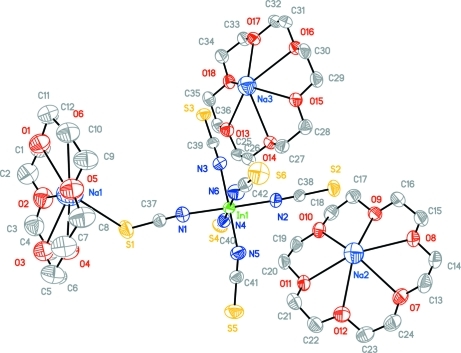
ORTEP drawing of the title complex with atomic numbering scheme and thermal ellipsoids at 30% probability level. H atoms and solvent molecules have been omitted for clarity.

**Fig. 2.**
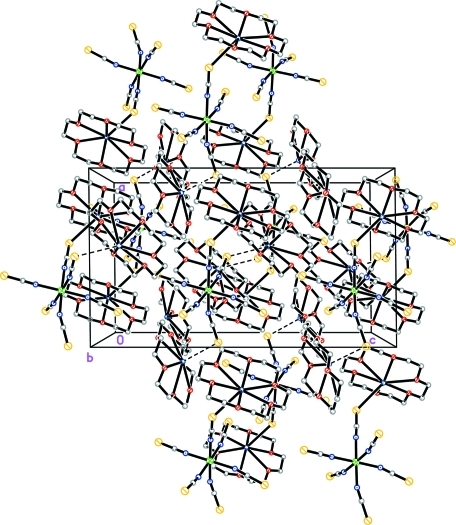
The crystal packing of (I), viewed along the a axis.

## supplementary crystallographic information

### Comment

Many interests have focused on crown ethers and their metal cations, because they can act as modules to form polymeric supramolecular structures with novel crystal engineering (Desai *et al.*, 2001). Now we report a complex in this paper, and present here the crystal structure of the title complex, (I).

The asymmetric unit of the complex consists of three [Na(18—C-6)]+ cations, one In(SCN)_6_3-anions and one and half molecules of dichloroethane. The In atom is coordinated with six N atoms. The average bond length of In—N is 2.190 Å and the mean bond angle of N—In—N is 178.1°; these are comparable with those observed in similar compounds (Mullica *et al.*, 1999). In all the crystallographically independent [Na(18—C-6)]+ cations, the Na+ ion is coordinated with the six O atoms of the crown ether moeity. The Na—O bond lengths varied from 2.842 (10) to 3.058 (5) Å and were longer than the similar complex [Na(18—C-6)]2[Zn(mnt)2] [mnt=1,2-dicyanoethene-1,2-dithiolate] and the other reported complexes (Han, *et al.*, 1987; Han, *et al.*, 1989). Additionally the Na+ ion is also coordinated with S atom of (SCN)-. The Na—S bond lengths were found to be in the range of 3.264 (9)–3.661 (5) Å which is found to be similar with the reported compound (Noth *et al.*, 2004).

### Experimental

NaSCN (3 mmol), InCl3 (0.5 mmol) and 15 ml dichloroethane were refluxed for 4 h in a 50 ml round-bottom flask. Then the 18-Crown-6 (3 mmol) was added slowly to the mixtures and the reaction mixture was refluxed for another 4 h. After cooling to room temperature, the mixture was filtered. The solid title complex, thus obtained, was recrystallized from ether. Elemental analysis: calculated for C45H78Cl3InN6Na3O18S6: C 36.68, H 5.33, N 5.70%; found: C 36.72,H 5.17, N 5.61%.

### Refinement

All H atoms were positioned geometrically and refined using a riding model with C—H =0.97 Å and *U*_iso_(H) = 1.2 *U*_eq_(C). Moreover, one of the solvent (CH2)2Cl2 is located on a center of symmetry.

### Crystal data

**Table:**

[Na(C_12_H_24_O_6_)]_2_[InNa(NCS)_6_(C_12_H_24_O_6_)]·1.5C_2_H_4_Cl_2_	*F*(000) = 3052
*M**_r_* = 1473.63	*D*_x_ = 1.372 Mg m^−^^3^
Monoclinic, *P*2_1_/*c*	Mo *K*α radiation, λ = 0.71073 Å
Hall symbol: -P 2ybc	Cell parameters from 6947 reflections
*a* = 13.7304 (15) Å	θ = 2.3–21.3°
*b* = 22.144 (2) Å	µ = 0.70 mm^−^^1^
*c* = 23.469 (3) Å	*T* = 298 K
β = 90.541 (2)°	Block, colourless
*V* = 7135.3 (13) Å^3^	0.29 × 0.27 × 0.25 mm
*Z* = 4	

### Data collection

**Table:**

Bruker SMART CCD area-detector diffractometer	12551 independent reflections
Radiation source: fine-focus sealed tube	6533 reflections with *I* > 2σ(*I*)
graphite	*R*_int_ = 0.051
φ and ω scans	θ_max_ = 25.0°, θ_min_ = 1.8°
Absorption correction: multi-scan (*SADABS*; Sheldrick, 1996)	*h* = −16→16
*T*_min_ = 0.823, *T*_max_ = 0.845	*k* = −26→23
37284 measured reflections	*l* = −25→27

### Refinement

**Table:**

Refinement on *F*^2^	Primary atom site location: structure-invariant direct methods
Least-squares matrix: full	Secondary atom site location: difference Fourier map
*R*[*F*^2^ > 2σ(*F*^2^)] = 0.071	Hydrogen site location: inferred from neighbouring sites
*wR*(*F*^2^) = 0.236	H-atom parameters constrained
*S* = 1.02	*w* = 1/[σ^2^(*F*_o_^2^) + (0.098*P*)^2^ + 21.6303*P*] where *P* = (*F*_o_^2^ + 2*F*_c_^2^)/3
12551 reflections	(Δ/σ)_max_ = 0.058
739 parameters	Δρ_max_ = 1.30 e Å^−^^3^
1314 restraints	Δρ_min_ = −1.09 e Å^−^^3^

### Special details

**Table:**

Geometry. All e.s.d.'s (except the e.s.d. in the dihedral angle between two l.s. planes) are estimated using the full covariance matrix. The cell e.s.d.'s are taken into account individually in the estimation of e.s.d.'s in distances, angles and torsion angles; correlations between e.s.d.'s in cell parameters are only used when they are defined by crystal symmetry. An approximate (isotropic) treatment of cell e.s.d.'s is used for estimating e.s.d.'s involving l.s. planes.
Refinement. Refinement of *F*^2^ against ALL reflections. The weighted *R*-factor *wR* and goodness of fit *S* are based on *F*^2^, conventional *R*-factors *R* are based on *F*, with *F* set to zero for negative *F*^2^. The threshold expression of *F*^2^ > σ(*F*^2^) is used only for calculating *R*-factors(gt) *etc*. and is not relevant to the choice of reflections for refinement. *R*-factors based on *F*^2^ are statistically about twice as large as those based on *F*, and *R*- factors based on ALL data will be even larger.

### Fractional atomic coordinates and isotropic or equivalent isotropic displacement parameters (Å^2^)

**Table:**

	*x*	*y*	*z*	*U*_iso_*/*U*_eq_	
In1	0.30721 (4)	0.73423 (3)	0.38104 (2)	0.0553 (2)	
Na1	0.7472 (6)	0.9014 (4)	0.4923 (3)	0.203 (3)	
Na2	−0.1166 (5)	0.6852 (3)	0.2889 (3)	0.162 (2)	
Na3	0.4318 (4)	0.4082 (3)	0.4100 (3)	0.152 (2)	
Cl1	0.6945 (2)	0.44940 (15)	0.43849 (15)	0.1233 (11)	
Cl2	0.9244 (3)	0.4261 (3)	0.4170 (3)	0.252 (3)	
Cl3	1.0881 (5)	0.0110 (4)	0.4316 (3)	0.264 (3)	
N1	0.4407 (5)	0.7876 (4)	0.3851 (3)	0.0803 (19)	
N2	0.1717 (5)	0.6806 (3)	0.3795 (3)	0.0716 (18)	
N3	0.3875 (5)	0.6568 (3)	0.4170 (3)	0.0729 (18)	
N4	0.3448 (5)	0.7004 (4)	0.2964 (3)	0.0727 (18)	
N5	0.2301 (6)	0.8104 (4)	0.3434 (3)	0.083 (2)	
N6	0.2678 (6)	0.7640 (4)	0.4667 (3)	0.082 (2)	
O1	0.9094 (6)	0.8232 (4)	0.5207 (4)	0.128 (2)	
O2	0.8882 (7)	0.8638 (5)	0.4086 (4)	0.140 (3)	
O3	0.7986 (7)	0.9793 (5)	0.3954 (4)	0.142 (3)	
O4	0.6676 (8)	1.0215 (5)	0.4803 (5)	0.150 (3)	
O5	0.6743 (7)	0.9661 (4)	0.5884 (4)	0.132 (3)	
O6	0.7702 (6)	0.8548 (4)	0.6045 (3)	0.115 (2)	
O7	−0.3078 (5)	0.7354 (3)	0.2561 (3)	0.100 (2)	
O8	−0.2943 (4)	0.6233 (4)	0.3113 (3)	0.0924 (18)	
O9	−0.1353 (5)	0.5539 (3)	0.2770 (3)	0.0798 (16)	
O10	0.0376 (4)	0.6133 (3)	0.2458 (3)	0.0796 (16)	
O11	0.0292 (4)	0.7332 (3)	0.2075 (3)	0.0802 (16)	
O12	−0.1376 (6)	0.8020 (3)	0.2319 (3)	0.104 (2)	
O13	0.4885 (5)	0.4402 (3)	0.2933 (3)	0.0834 (17)	
O14	0.2988 (4)	0.4777 (3)	0.3279 (3)	0.0767 (16)	
O15	0.2319 (4)	0.4397 (3)	0.4352 (3)	0.0798 (16)	
O16	0.2952 (4)	0.3263 (3)	0.4770 (2)	0.0755 (15)	
O17	0.4762 (4)	0.2848 (3)	0.4379 (3)	0.0756 (16)	
O18	0.5467 (4)	0.3276 (3)	0.3317 (3)	0.0791 (16)	
S1	0.5860 (2)	0.87048 (15)	0.39347 (13)	0.1094 (10)	
S2	−0.00642 (17)	0.63665 (14)	0.41181 (10)	0.0845 (8)	
S3	0.50512 (17)	0.56055 (10)	0.44467 (10)	0.0693 (6)	
S4	0.3969 (2)	0.63856 (12)	0.19996 (10)	0.0814 (7)	
S5	0.1789 (2)	0.92120 (13)	0.30143 (14)	0.1068 (10)	
S6	0.1800 (3)	0.79117 (18)	0.56726 (13)	0.1210 (11)	
C1	0.9412 (11)	0.7928 (7)	0.4752 (6)	0.142 (4)	
H1A	0.8906	0.7650	0.4629	0.170*	
H1B	0.9972	0.7688	0.4867	0.170*	
C2	0.9683 (11)	0.8296 (7)	0.4274 (6)	0.145 (4)	
H2A	1.0210	0.8565	0.4385	0.174*	
H2B	0.9910	0.8041	0.3967	0.174*	
C3	0.9010 (11)	0.9021 (7)	0.3628 (6)	0.148 (4)	
H3A	0.9149	0.8780	0.3294	0.178*	
H3B	0.9575	0.9273	0.3704	0.178*	
C4	0.8179 (11)	0.9414 (7)	0.3499 (6)	0.138 (3)	
H4A	0.8319	0.9654	0.3164	0.165*	
H4B	0.7610	0.9168	0.3417	0.165*	
C5	0.7243 (12)	1.0193 (7)	0.3858 (7)	0.147 (4)	
H5A	0.6673	0.9974	0.3726	0.176*	
H5B	0.7431	1.0471	0.3560	0.176*	
C6	0.6997 (12)	1.0531 (7)	0.4362 (7)	0.153 (4)	
H6A	0.7570	1.0753	0.4484	0.184*	
H6B	0.6502	1.0824	0.4258	0.184*	
C7	0.6453 (12)	1.0517 (7)	0.5299 (6)	0.151 (4)	
H7A	0.5962	1.0820	0.5218	0.181*	
H7B	0.7031	1.0723	0.5440	0.181*	
C8	0.6093 (11)	1.0107 (7)	0.5742 (6)	0.156 (4)	
H8A	0.5946	1.0340	0.6080	0.188*	
H8B	0.5492	0.9922	0.5609	0.188*	
C9	0.6517 (12)	0.9260 (7)	0.6302 (6)	0.138 (4)	
H9A	0.6008	0.8992	0.6163	0.166*	
H9B	0.6262	0.9479	0.6626	0.166*	
C10	0.7340 (10)	0.8902 (7)	0.6487 (5)	0.126 (3)	
H10A	0.7851	0.9167	0.6627	0.151*	
H10B	0.7144	0.8642	0.6799	0.151*	
C11	0.8558 (10)	0.8282 (6)	0.6172 (5)	0.121 (3)	
H11A	0.8480	0.8030	0.6507	0.145*	
H11B	0.9036	0.8591	0.6263	0.145*	
C12	0.8920 (10)	0.7908 (6)	0.5697 (6)	0.120 (3)	
H12A	0.9519	0.7712	0.5816	0.144*	
H12B	0.8446	0.7596	0.5612	0.144*	
C13	−0.3747 (8)	0.7165 (6)	0.2963 (6)	0.112 (3)	
H13A	−0.4377	0.7341	0.2875	0.134*	
H13B	−0.3544	0.7309	0.3335	0.134*	
C14	−0.3842 (7)	0.6508 (6)	0.2981 (5)	0.104 (3)	
H14A	−0.4318	0.6398	0.3265	0.125*	
H14B	−0.4075	0.6363	0.2614	0.125*	
C15	−0.2972 (8)	0.5604 (5)	0.3083 (5)	0.098 (3)	
H15A	−0.3202	0.5482	0.2708	0.118*	
H15B	−0.3425	0.5450	0.3362	0.118*	
C16	−0.2021 (8)	0.5351 (5)	0.3188 (5)	0.094 (3)	
H16A	−0.1789	0.5476	0.3561	0.113*	
H16B	−0.2065	0.4914	0.3186	0.113*	
C17	−0.0428 (7)	0.5282 (5)	0.2839 (4)	0.087 (2)	
H17A	−0.0474	0.4846	0.2811	0.104*	
H17B	−0.0168	0.5383	0.3213	0.104*	
C18	0.0219 (8)	0.5511 (5)	0.2401 (5)	0.091 (3)	
H18A	0.0838	0.5301	0.2426	0.109*	
H18B	−0.0063	0.5429	0.2029	0.109*	
C19	0.1053 (6)	0.6377 (5)	0.2085 (4)	0.088 (2)	
H19A	0.0830	0.6322	0.1695	0.105*	
H19B	0.1672	0.6171	0.2130	0.105*	
C20	0.1177 (6)	0.7029 (5)	0.2207 (4)	0.082 (2)	
H20A	0.1344	0.7087	0.2606	0.098*	
H20B	0.1701	0.7192	0.1978	0.098*	
C21	0.0328 (8)	0.7953 (5)	0.2193 (5)	0.098 (3)	
H21A	0.0872	0.8135	0.1996	0.117*	
H21B	0.0421	0.8016	0.2599	0.117*	
C22	−0.0581 (8)	0.8234 (5)	0.2006 (5)	0.100 (3)	
H22A	−0.0532	0.8668	0.2051	0.121*	
H22B	−0.0689	0.8149	0.1605	0.121*	
C23	−0.2296 (8)	0.8175 (5)	0.2089 (5)	0.117 (3)	
H23A	−0.2375	0.7989	0.1717	0.140*	
H23B	−0.2336	0.8609	0.2041	0.140*	
C24	−0.3076 (8)	0.7971 (6)	0.2464 (6)	0.118 (3)	
H24A	−0.3012	0.8178	0.2826	0.142*	
H24B	−0.3698	0.8086	0.2296	0.142*	
C25	0.4223 (8)	0.4746 (5)	0.2598 (4)	0.088 (3)	
H25A	0.3808	0.4477	0.2378	0.106*	
H25B	0.4582	0.4997	0.2334	0.106*	
C26	0.3622 (8)	0.5127 (5)	0.2961 (4)	0.086 (2)	
H26A	0.4037	0.5361	0.3215	0.104*	
H26B	0.3252	0.5408	0.2726	0.104*	
C27	0.2385 (7)	0.5138 (4)	0.3632 (4)	0.083 (2)	
H27A	0.1987	0.5402	0.3397	0.100*	
H27B	0.2788	0.5387	0.3878	0.100*	
C28	0.1748 (7)	0.4747 (5)	0.3984 (4)	0.085 (2)	
H28A	0.1307	0.4996	0.4202	0.101*	
H28B	0.1363	0.4486	0.3738	0.101*	
C29	0.1775 (7)	0.4036 (5)	0.4731 (4)	0.086 (2)	
H29A	0.1360	0.3764	0.4515	0.103*	
H29B	0.1362	0.4292	0.4961	0.103*	
C30	0.2433 (7)	0.3683 (5)	0.5106 (4)	0.084 (2)	
H30A	0.2885	0.3951	0.5300	0.101*	
H30B	0.2056	0.3470	0.5390	0.101*	
C31	0.3565 (7)	0.2886 (4)	0.5094 (4)	0.080 (2)	
H31A	0.3177	0.2639	0.5346	0.096*	
H31B	0.4002	0.3130	0.5325	0.096*	
C32	0.4138 (7)	0.2493 (4)	0.4711 (4)	0.084 (2)	
H32A	0.4518	0.2209	0.4936	0.101*	
H32B	0.3702	0.2266	0.4465	0.101*	
C33	0.5335 (7)	0.2495 (4)	0.3996 (4)	0.084 (2)	
H33A	0.4912	0.2282	0.3730	0.101*	
H33B	0.5712	0.2199	0.4207	0.101*	
C34	0.5994 (7)	0.2901 (5)	0.3681 (5)	0.089 (3)	
H34A	0.6365	0.3144	0.3949	0.107*	
H34B	0.6449	0.2662	0.3462	0.107*	
C35	0.6077 (7)	0.3625 (5)	0.2963 (4)	0.087 (2)	
H35A	0.6468	0.3360	0.2729	0.105*	
H35B	0.6514	0.3867	0.3196	0.105*	
C36	0.5472 (7)	0.4024 (5)	0.2595 (4)	0.089 (3)	
H36A	0.5890	0.4268	0.2357	0.107*	
H36B	0.5062	0.3779	0.2347	0.107*	
C37	0.5002 (6)	0.8222 (4)	0.3897 (3)	0.063 (2)	
C38	0.0984 (6)	0.6634 (4)	0.3929 (3)	0.0566 (18)	
C39	0.4365 (5)	0.6167 (4)	0.4286 (3)	0.0539 (18)	
C40	0.3667 (6)	0.6748 (4)	0.2564 (3)	0.0591 (19)	
C41	0.2089 (6)	0.8567 (4)	0.3265 (4)	0.067 (2)	
C42	0.2309 (6)	0.7754 (4)	0.5082 (4)	0.070 (2)	
C43	0.7700 (9)	0.4016 (6)	0.4773 (5)	0.132 (4)	
H43A	0.7925	0.4234	0.5108	0.159*	
H43B	0.7310	0.3680	0.4905	0.159*	
C44	0.8533 (9)	0.3777 (6)	0.4492 (7)	0.145 (5)	
H44A	0.8309	0.3485	0.4212	0.174*	
H44B	0.8922	0.3559	0.4771	0.174*	
C45	0.9901 (19)	0.0294 (8)	0.4813 (9)	0.248 (10)	
H45A	0.9261	0.0297	0.4635	0.298*	
H45B	1.0013	0.0667	0.5021	0.298*	

### Atomic displacement parameters (Å^2^)

**Table:**

	*U*^11^	*U*^22^	*U*^33^	*U*^12^	*U*^13^	*U*^23^
In1	0.0498 (3)	0.0591 (4)	0.0572 (3)	0.0037 (3)	0.0033 (2)	0.0056 (3)
Na1	0.228 (8)	0.224 (8)	0.158 (6)	0.013 (7)	0.010 (5)	0.010 (5)
Na2	0.147 (5)	0.169 (6)	0.169 (5)	−0.002 (4)	0.010 (4)	0.003 (4)
Na3	0.148 (5)	0.157 (5)	0.149 (5)	−0.004 (4)	0.010 (4)	0.000 (4)
Cl1	0.098 (2)	0.118 (2)	0.155 (3)	0.0274 (18)	0.042 (2)	0.030 (2)
Cl2	0.096 (3)	0.355 (6)	0.306 (6)	0.030 (3)	0.034 (3)	0.207 (5)
Cl3	0.251 (6)	0.306 (7)	0.236 (5)	−0.121 (5)	0.016 (5)	−0.009 (5)
N1	0.072 (4)	0.082 (4)	0.087 (4)	−0.010 (4)	0.002 (4)	0.002 (4)
N2	0.052 (4)	0.085 (4)	0.078 (4)	−0.004 (3)	0.003 (3)	0.007 (4)
N3	0.067 (4)	0.075 (4)	0.076 (4)	0.003 (4)	−0.006 (3)	0.008 (4)
N4	0.063 (4)	0.092 (5)	0.063 (4)	0.001 (4)	0.003 (3)	0.001 (4)
N5	0.075 (4)	0.088 (5)	0.085 (5)	0.014 (4)	0.002 (4)	0.015 (4)
N6	0.093 (5)	0.088 (5)	0.067 (4)	0.006 (4)	0.012 (4)	0.000 (4)
O1	0.129 (5)	0.132 (6)	0.122 (6)	0.024 (5)	−0.001 (5)	−0.006 (5)
O2	0.133 (6)	0.168 (7)	0.119 (6)	−0.009 (5)	0.022 (5)	−0.003 (5)
O3	0.144 (6)	0.155 (6)	0.126 (6)	−0.020 (5)	−0.015 (5)	0.021 (5)
O4	0.177 (7)	0.141 (6)	0.130 (6)	0.010 (6)	−0.009 (6)	0.025 (5)
O5	0.154 (6)	0.137 (6)	0.106 (5)	0.019 (5)	0.005 (5)	−0.001 (5)
O6	0.130 (5)	0.122 (5)	0.093 (5)	−0.002 (5)	−0.008 (4)	0.016 (4)
O7	0.076 (4)	0.097 (4)	0.127 (5)	0.026 (4)	0.015 (4)	0.000 (4)
O8	0.065 (4)	0.112 (5)	0.100 (4)	−0.007 (4)	0.010 (3)	0.011 (4)
O9	0.082 (4)	0.080 (4)	0.077 (4)	−0.008 (3)	0.000 (3)	0.005 (3)
O10	0.065 (3)	0.093 (4)	0.081 (4)	0.006 (3)	0.016 (3)	−0.010 (3)
O11	0.068 (3)	0.094 (4)	0.079 (4)	−0.014 (3)	0.003 (3)	−0.002 (3)
O12	0.100 (5)	0.084 (4)	0.127 (5)	0.005 (4)	−0.001 (4)	0.008 (4)
O13	0.086 (4)	0.096 (4)	0.068 (4)	−0.006 (3)	0.010 (3)	−0.001 (3)
O14	0.079 (4)	0.069 (4)	0.082 (4)	−0.005 (3)	0.002 (3)	0.008 (3)
O15	0.066 (3)	0.085 (4)	0.088 (4)	0.002 (3)	0.009 (3)	0.012 (3)
O16	0.072 (4)	0.081 (4)	0.073 (3)	−0.003 (3)	0.005 (3)	0.003 (3)
O17	0.083 (4)	0.063 (3)	0.081 (4)	−0.003 (3)	0.006 (3)	0.004 (3)
O18	0.067 (3)	0.086 (4)	0.085 (4)	−0.004 (3)	0.017 (3)	−0.003 (3)
S1	0.113 (2)	0.114 (2)	0.101 (2)	−0.0523 (19)	−0.0150 (17)	0.0134 (17)
S2	0.0620 (14)	0.122 (2)	0.0696 (14)	−0.0190 (14)	0.0099 (11)	0.0070 (14)
S3	0.0766 (15)	0.0585 (13)	0.0727 (14)	0.0089 (11)	−0.0106 (11)	0.0024 (11)
S4	0.1029 (19)	0.0797 (17)	0.0618 (14)	−0.0034 (14)	0.0159 (13)	−0.0006 (12)
S5	0.116 (2)	0.0702 (17)	0.134 (3)	0.0247 (16)	0.0057 (19)	0.0218 (17)
S6	0.134 (3)	0.148 (3)	0.0821 (19)	0.008 (2)	0.0284 (18)	−0.0318 (19)
C1	0.145 (7)	0.150 (8)	0.130 (7)	0.010 (7)	0.009 (7)	−0.018 (7)
C2	0.140 (7)	0.160 (8)	0.135 (7)	0.021 (7)	0.015 (7)	−0.014 (7)
C3	0.152 (8)	0.169 (8)	0.124 (7)	−0.022 (7)	0.014 (7)	0.006 (7)
C4	0.145 (7)	0.157 (8)	0.112 (7)	−0.023 (7)	0.012 (6)	0.021 (6)
C5	0.162 (8)	0.145 (8)	0.134 (7)	−0.007 (7)	−0.002 (7)	0.053 (7)
C6	0.179 (8)	0.142 (8)	0.138 (8)	−0.001 (7)	−0.002 (7)	0.042 (7)
C7	0.172 (8)	0.149 (8)	0.132 (7)	0.028 (7)	0.000 (7)	0.006 (7)
C8	0.174 (8)	0.151 (8)	0.144 (8)	0.041 (7)	0.002 (7)	0.013 (7)
C9	0.165 (8)	0.147 (8)	0.102 (7)	0.017 (7)	0.012 (7)	0.014 (6)
C10	0.148 (7)	0.144 (7)	0.087 (6)	0.016 (7)	0.012 (6)	0.000 (6)
C11	0.122 (7)	0.126 (7)	0.113 (7)	0.018 (6)	−0.023 (6)	0.022 (6)
C12	0.118 (7)	0.128 (7)	0.113 (7)	0.013 (6)	−0.015 (6)	0.021 (6)
C13	0.071 (5)	0.123 (7)	0.142 (7)	0.013 (5)	0.020 (5)	−0.014 (6)
C14	0.061 (5)	0.132 (7)	0.119 (6)	0.003 (5)	0.017 (5)	0.018 (6)
C15	0.086 (6)	0.118 (6)	0.092 (6)	−0.017 (5)	0.009 (5)	0.017 (5)
C16	0.093 (6)	0.092 (6)	0.098 (6)	−0.014 (5)	0.007 (5)	0.014 (5)
C17	0.090 (6)	0.082 (5)	0.089 (5)	0.008 (5)	−0.004 (5)	−0.005 (5)
C18	0.082 (5)	0.093 (6)	0.097 (6)	0.019 (5)	0.000 (5)	−0.016 (5)
C19	0.057 (5)	0.122 (6)	0.086 (5)	0.007 (5)	0.015 (4)	−0.001 (5)
C20	0.060 (4)	0.107 (5)	0.079 (5)	−0.013 (4)	0.010 (4)	0.008 (5)
C21	0.096 (6)	0.091 (6)	0.106 (6)	−0.022 (5)	0.007 (5)	0.003 (5)
C22	0.101 (6)	0.083 (6)	0.117 (6)	−0.012 (5)	0.009 (5)	0.008 (5)
C23	0.106 (6)	0.094 (6)	0.150 (7)	0.031 (6)	0.003 (6)	0.007 (6)
C24	0.094 (6)	0.100 (6)	0.160 (7)	0.035 (6)	0.007 (6)	0.001 (6)
C25	0.102 (6)	0.088 (6)	0.074 (5)	−0.001 (5)	0.005 (5)	0.020 (5)
C26	0.097 (5)	0.081 (5)	0.082 (5)	−0.009 (5)	0.002 (5)	0.020 (4)
C27	0.083 (5)	0.068 (5)	0.098 (6)	0.007 (4)	−0.006 (5)	0.000 (5)
C28	0.075 (5)	0.081 (5)	0.098 (6)	0.002 (5)	0.006 (5)	−0.006 (5)
C29	0.065 (5)	0.091 (6)	0.101 (6)	−0.001 (4)	0.021 (4)	0.002 (5)
C30	0.067 (5)	0.099 (6)	0.088 (5)	−0.004 (5)	0.025 (4)	0.007 (5)
C31	0.086 (5)	0.080 (5)	0.075 (5)	−0.006 (5)	0.002 (4)	0.017 (4)
C32	0.089 (5)	0.079 (5)	0.084 (5)	−0.001 (4)	0.010 (5)	0.018 (4)
C33	0.083 (5)	0.066 (5)	0.103 (6)	0.010 (4)	0.017 (5)	0.001 (4)
C34	0.084 (5)	0.077 (5)	0.106 (6)	0.004 (5)	0.014 (5)	0.003 (5)
C35	0.082 (5)	0.096 (6)	0.084 (5)	−0.009 (5)	0.025 (5)	−0.003 (5)
C36	0.089 (5)	0.101 (6)	0.077 (5)	−0.020 (5)	0.020 (5)	−0.001 (5)
C37	0.063 (2)	0.063 (2)	0.063 (2)	−0.0005 (10)	0.0007 (10)	0.0000 (10)
C38	0.048 (4)	0.070 (5)	0.051 (4)	0.003 (4)	0.000 (3)	0.006 (4)
C39	0.053 (4)	0.058 (5)	0.051 (4)	0.005 (4)	−0.001 (3)	0.005 (4)
C40	0.053 (4)	0.068 (5)	0.057 (4)	−0.004 (4)	0.001 (4)	0.011 (4)
C41	0.063 (5)	0.070 (5)	0.070 (5)	0.009 (4)	0.002 (4)	0.005 (4)
C42	0.070 (2)	0.070 (2)	0.069 (2)	0.0006 (10)	0.0006 (10)	−0.0002 (10)
C43	0.115 (9)	0.151 (10)	0.131 (9)	0.017 (8)	0.024 (7)	0.067 (8)
C44	0.109 (9)	0.135 (10)	0.190 (11)	0.019 (8)	−0.002 (9)	0.060 (9)
C45	0.239 (12)	0.255 (13)	0.251 (13)	−0.012 (10)	0.017 (9)	−0.019 (9)

### Geometric parameters (Å, °)

**Table:**

In1—N5	2.174 (8)	C5—H5A	0.9700
In1—N1	2.181 (8)	C5—H5B	0.9700
In1—N6	2.187 (7)	C6—H6A	0.9700
In1—N4	2.189 (8)	C6—H6B	0.9700
In1—N3	2.203 (8)	C7—C8	1.469 (14)
In1—N2	2.207 (7)	C7—H7A	0.9700
Na1—O6	2.842 (10)	C7—H7B	0.9700
Na1—O5	2.860 (12)	C8—H8A	0.9700
Na1—O4	2.888 (13)	C8—H8B	0.9700
Na1—O2	2.894 (12)	C9—C10	1.445 (17)
Na1—O1	2.893 (12)	C9—H9A	0.9700
Na1—O3	2.946 (12)	C9—H9B	0.9700
Na1—S1	3.264 (9)	C10—H10A	0.9700
Na2—O10	2.844 (9)	C10—H10B	0.9700
Na2—O8	2.852 (9)	C11—C12	1.480 (16)
Na2—O12	2.925 (10)	C11—H11A	0.9700
Na2—O9	2.932 (9)	C11—H11B	0.9700
Na2—O7	2.947 (9)	C12—H12A	0.9700
Na2—O11	2.977 (9)	C12—H12B	0.9700
Na3—O17	2.873 (9)	C13—C14	1.460 (15)
Na3—O15	2.899 (8)	C13—H13A	0.9700
Na3—O13	2.942 (8)	C13—H13B	0.9700
Na3—O18	3.016 (8)	C14—H14A	0.9700
Na3—O16	3.055 (8)	C14—H14B	0.9700
Na3—O14	3.058 (9)	C15—C16	1.439 (13)
Cl1—C43	1.733 (12)	C15—H15A	0.9700
Cl2—C44	1.639 (11)	C15—H15B	0.9700
Cl3—C45	1.84 (3)	C16—H16A	0.9700
N1—C37	1.126 (10)	C16—H16B	0.9700
N2—C38	1.124 (9)	C17—C18	1.456 (13)
N3—C39	1.144 (9)	C17—H17A	0.9700
N4—C40	1.141 (10)	C17—H17B	0.9700
N5—C41	1.137 (10)	C18—H18A	0.9700
N6—C42	1.132 (10)	C18—H18B	0.9700
O1—C1	1.339 (14)	C19—C20	1.481 (11)
O1—C12	1.378 (14)	C19—H19A	0.9700
O2—C3	1.382 (15)	C19—H19B	0.9700
O2—C2	1.403 (16)	C20—H20A	0.9700
O3—C5	1.367 (16)	C20—H20B	0.9700
O3—C4	1.387 (13)	C21—C22	1.460 (14)
O4—C6	1.329 (15)	C21—H21A	0.9700
O4—C7	1.379 (12)	C21—H21B	0.9700
O5—C9	1.361 (14)	C22—H22A	0.9700
O5—C8	1.370 (12)	C22—H22B	0.9700
O6—C11	1.345 (13)	C23—C24	1.463 (13)
O6—C10	1.395 (13)	C23—H23A	0.9700
O7—C13	1.387 (13)	C23—H23B	0.9700
O7—C24	1.386 (13)	C24—H24A	0.9700
O8—C15	1.395 (12)	C24—H24B	0.9700
O8—C14	1.409 (11)	C25—C26	1.460 (13)
O9—C17	1.399 (11)	C25—H25A	0.9700
O9—C16	1.413 (11)	C25—H25B	0.9700
O10—C19	1.391 (9)	C26—H26A	0.9700
O10—C18	1.400 (11)	C26—H26B	0.9700
O11—C21	1.402 (12)	C27—C28	1.487 (13)
O11—C20	1.420 (10)	C27—H27A	0.9700
O12—C22	1.403 (12)	C27—H27B	0.9700
O12—C23	1.412 (11)	C28—H28A	0.9700
O13—C36	1.411 (11)	C28—H28B	0.9700
O13—C25	1.419 (11)	C29—C30	1.479 (13)
O14—C26	1.388 (10)	C29—H29A	0.9700
O14—C27	1.422 (10)	C29—H29B	0.9700
O15—C28	1.396 (11)	C30—H30A	0.9700
O15—C29	1.412 (10)	C30—H30B	0.9700
O16—C31	1.405 (10)	C31—C32	1.482 (13)
O16—C30	1.415 (10)	C31—H31A	0.9700
O17—C32	1.403 (10)	C31—H31B	0.9700
O17—C33	1.433 (10)	C32—H32A	0.9700
O18—C34	1.390 (11)	C32—H32B	0.9700
O18—C35	1.414 (10)	C33—C34	1.478 (13)
S1—C37	1.593 (9)	C33—H33A	0.9700
S2—C38	1.622 (8)	C33—H33B	0.9700
S3—C39	1.603 (8)	C34—H34A	0.9700
S4—C40	1.606 (9)	C34—H34B	0.9700
S5—C41	1.597 (10)	C35—C36	1.486 (13)
S6—C42	1.596 (10)	C35—H35A	0.9700
C1—C2	1.439 (18)	C35—H35B	0.9700
C1—H1A	0.9700	C36—H36A	0.9700
C1—H1B	0.9700	C36—H36B	0.9700
C2—H2A	0.9700	C43—C44	1.429 (13)
C2—H2B	0.9700	C43—H43A	0.9700
C3—C4	1.463 (14)	C43—H43B	0.9700
C3—H3A	0.9700	C44—H44A	0.9700
C3—H3B	0.9700	C44—H44B	0.9700
C4—H4A	0.9700	C45—C45^i^	1.592 (19)
C4—H4B	0.9700	C45—H45A	0.9700
C5—C6	1.441 (19)	C45—H45B	0.9700
			
N5—In1—N1	90.2 (3)	C10—C9—H9A	109.0
N5—In1—N6	91.0 (3)	O5—C9—H9B	109.0
N1—In1—N6	90.7 (3)	C10—C9—H9B	109.0
N5—In1—N4	90.8 (3)	H9A—C9—H9B	107.8
N1—In1—N4	91.1 (3)	O6—C10—C9	111.5 (11)
N6—In1—N4	177.5 (3)	O6—C10—H10A	109.3
N5—In1—N3	178.4 (3)	C9—C10—H10A	109.3
N1—In1—N3	89.3 (3)	O6—C10—H10B	109.3
N6—In1—N3	90.6 (3)	C9—C10—H10B	109.3
N4—In1—N3	87.7 (3)	H10A—C10—H10B	108.0
N5—In1—N2	90.2 (3)	O6—C11—C12	112.2 (11)
N1—In1—N2	178.4 (3)	O6—C11—H11A	109.2
N6—In1—N2	87.7 (3)	C12—C11—H11A	109.2
N4—In1—N2	90.4 (3)	O6—C11—H11B	109.2
N3—In1—N2	90.3 (3)	C12—C11—H11B	109.2
O6—Na1—O5	59.2 (3)	H11A—C11—H11B	107.9
O6—Na1—O4	117.6 (4)	O1—C12—C11	113.6 (11)
O5—Na1—O4	58.7 (3)	O1—C12—H12A	108.9
O6—Na1—O2	117.1 (4)	C11—C12—H12A	108.8
O5—Na1—O2	157.1 (4)	O1—C12—H12B	108.8
O4—Na1—O2	117.0 (4)	C11—C12—H12B	108.9
O6—Na1—O1	59.4 (3)	H12A—C12—H12B	107.7
O5—Na1—O1	113.1 (4)	O7—C13—C14	112.4 (10)
O4—Na1—O1	149.7 (4)	O7—C13—H13A	109.1
O2—Na1—O1	57.7 (3)	C14—C13—H13A	109.1
O6—Na1—O3	154.5 (4)	O7—C13—H13B	109.1
O5—Na1—O3	113.8 (4)	C14—C13—H13B	109.1
O4—Na1—O3	58.6 (4)	H13A—C13—H13B	107.9
O2—Na1—O3	58.5 (4)	O8—C14—C13	111.0 (9)
O1—Na1—O3	109.8 (4)	O8—C14—H14A	109.4
O6—Na1—S1	130.6 (3)	C13—C14—H14A	109.5
O5—Na1—S1	115.2 (3)	O8—C14—H14B	109.4
O4—Na1—S1	82.5 (3)	C13—C14—H14B	109.4
O2—Na1—S1	84.8 (3)	H14A—C14—H14B	108.0
O1—Na1—S1	123.6 (3)	O8—C15—C16	110.8 (9)
O3—Na1—S1	74.9 (3)	O8—C15—H15A	109.5
O10—Na2—O8	116.0 (3)	C16—C15—H15A	109.5
O10—Na2—O12	113.8 (3)	O8—C15—H15B	109.5
O8—Na2—O12	115.4 (3)	C16—C15—H15B	109.5
O10—Na2—O9	58.3 (2)	H15A—C15—H15B	108.1
O8—Na2—O9	57.8 (2)	O9—C16—C15	111.1 (9)
O12—Na2—O9	145.6 (3)	O9—C16—H16A	109.4
O10—Na2—O7	141.5 (3)	C15—C16—H16A	109.4
O8—Na2—O7	57.8 (2)	O9—C16—H16B	109.4
O12—Na2—O7	57.6 (2)	C15—C16—H16B	109.4
O9—Na2—O7	105.8 (3)	H16A—C16—H16B	108.0
O10—Na2—O11	57.6 (2)	O9—C17—C18	109.6 (8)
O8—Na2—O11	150.7 (3)	O9—C17—H17A	109.8
O12—Na2—O11	56.9 (2)	C18—C17—H17A	109.8
O9—Na2—O11	110.6 (3)	O9—C17—H17B	109.8
O7—Na2—O11	107.5 (3)	C18—C17—H17B	109.8
O10—Na2—S2	78.4 (2)	H17A—C17—H17B	108.2
O8—Na2—S2	93.8 (2)	O10—C18—C17	111.7 (8)
O12—Na2—S2	134.8 (3)	O10—C18—H18A	109.3
O9—Na2—S2	78.8 (2)	C17—C18—H18A	109.3
O7—Na2—S2	136.5 (3)	O10—C18—H18B	109.3
O11—Na2—S2	111.0 (2)	C17—C18—H18B	109.3
O17—Na3—O15	112.4 (3)	H18A—C18—H18B	107.9
O17—Na3—O13	112.6 (3)	O10—C19—C20	109.6 (8)
O15—Na3—O13	113.1 (3)	O10—C19—H19A	109.8
O17—Na3—O18	57.7 (2)	C20—C19—H19A	109.8
O15—Na3—O18	140.2 (3)	O10—C19—H19B	109.8
O13—Na3—O18	55.3 (2)	C20—C19—H19B	109.8
O17—Na3—O16	56.5 (2)	H19A—C19—H19B	108.2
O15—Na3—O16	56.7 (2)	O11—C20—C19	108.8 (8)
O13—Na3—O16	142.3 (3)	O11—C20—H20A	109.9
O18—Na3—O16	106.9 (3)	C19—C20—H20A	109.9
O17—Na3—O14	138.3 (3)	O11—C20—H20B	109.9
O15—Na3—O14	56.4 (2)	C19—C20—H20B	109.9
O13—Na3—O14	56.9 (2)	H20A—C20—H20B	108.3
O18—Na3—O14	103.1 (2)	O11—C21—C22	109.3 (9)
O16—Na3—O14	104.9 (2)	O11—C21—H21A	109.8
O17—Na3—S3	141.1 (2)	C22—C21—H21A	109.8
O15—Na3—S3	89.5 (2)	O11—C21—H21B	109.8
O13—Na3—S3	84.8 (2)	C22—C21—H21B	109.8
O18—Na3—S3	122.9 (2)	H21A—C21—H21B	108.3
O16—Na3—S3	127.6 (2)	O12—C22—C21	111.4 (9)
O14—Na3—S3	80.49 (19)	O12—C22—H22A	109.3
C37—N1—In1	169.2 (8)	C21—C22—H22A	109.3
C38—N2—In1	159.2 (7)	O12—C22—H22B	109.3
C39—N3—In1	170.2 (7)	C21—C22—H22B	109.3
C40—N4—In1	169.7 (7)	H22A—C22—H22B	108.0
C41—N5—In1	164.5 (8)	O12—C23—C24	110.6 (10)
C42—N6—In1	167.3 (8)	O12—C23—H23A	109.5
C1—O1—C12	117.6 (12)	C24—C23—H23A	109.5
C1—O1—Na1	112.0 (9)	O12—C23—H23B	109.5
C12—O1—Na1	111.4 (8)	C24—C23—H23B	109.5
C3—O2—C2	118.1 (12)	H23A—C23—H23B	108.1
C3—O2—Na1	116.2 (9)	O7—C24—C23	113.9 (10)
C2—O2—Na1	117.9 (8)	O7—C24—H24A	108.8
C5—O3—C4	114.4 (12)	C23—C24—H24A	108.8
C5—O3—Na1	108.8 (8)	O7—C24—H24B	108.8
C4—O3—Na1	106.8 (8)	C23—C24—H24B	108.8
C6—O4—C7	118.7 (13)	H24A—C24—H24B	107.7
C6—O4—Na1	115.6 (10)	O13—C25—C26	110.5 (8)
C7—O4—Na1	116.8 (8)	O13—C25—H25A	109.5
C9—O5—C8	119.5 (12)	C26—C25—H25A	109.5
C9—O5—Na1	109.0 (8)	O13—C25—H25B	109.5
C8—O5—Na1	113.7 (8)	C26—C25—H25B	109.5
C11—O6—C10	113.4 (10)	H25A—C25—H25B	108.1
C11—O6—Na1	116.9 (8)	O14—C26—C25	110.5 (8)
C10—O6—Na1	116.6 (7)	O14—C26—H26A	109.5
C13—O7—C24	114.3 (9)	C25—C26—H26A	109.5
C13—O7—Na2	107.6 (7)	O14—C26—H26B	109.5
C24—O7—Na2	114.3 (7)	C25—C26—H26B	109.5
C15—O8—C14	113.3 (8)	H26A—C26—H26B	108.1
C15—O8—Na2	119.6 (6)	O14—C27—C28	110.2 (8)
C14—O8—Na2	120.1 (6)	O14—C27—H27A	109.6
C17—O9—C16	113.2 (7)	C28—C27—H27A	109.6
C17—O9—Na2	108.3 (5)	O14—C27—H27B	109.6
C16—O9—Na2	106.4 (6)	C28—C27—H27B	109.6
C19—O10—C18	115.2 (7)	H27A—C27—H27B	108.1
C19—O10—Na2	120.6 (6)	O15—C28—C27	109.8 (8)
C18—O10—Na2	118.0 (5)	O15—C28—H28A	109.7
C21—O11—C20	113.1 (7)	C27—C28—H28A	109.7
C21—O11—Na2	104.2 (6)	O15—C28—H28B	109.7
C20—O11—Na2	105.7 (5)	C27—C28—H28B	109.7
C22—O12—C23	114.5 (9)	H28A—C28—H28B	108.2
C22—O12—Na2	117.7 (6)	O15—C29—C30	110.5 (7)
C23—O12—Na2	118.2 (7)	O15—C29—H29A	109.5
C36—O13—C25	112.0 (7)	C30—C29—H29A	109.5
C36—O13—Na3	122.5 (6)	O15—C29—H29B	109.5
C25—O13—Na3	118.1 (5)	C30—C29—H29B	109.6
C26—O14—C27	111.8 (7)	H29A—C29—H29B	108.1
C26—O14—Na3	104.3 (5)	O16—C30—C29	108.9 (8)
C27—O14—Na3	105.2 (5)	O16—C30—H30A	109.9
C28—O15—C29	114.0 (7)	C29—C30—H30A	109.9
C28—O15—Na3	122.2 (5)	O16—C30—H30B	109.9
C29—O15—Na3	119.9 (5)	C29—C30—H30B	109.9
C31—O16—C30	113.0 (7)	H30A—C30—H30B	108.3
C31—O16—Na3	105.3 (5)	O16—C31—C32	109.8 (8)
C30—O16—Na3	102.3 (5)	O16—C31—H31A	109.7
C32—O17—C33	112.6 (7)	C32—C31—H31A	109.7
C32—O17—Na3	122.0 (5)	O16—C31—H31B	109.7
C33—O17—Na3	119.4 (5)	C32—C31—H31B	109.7
C34—O18—C35	112.3 (7)	H31A—C31—H31B	108.2
C34—O18—Na3	104.6 (5)	O17—C32—C31	109.8 (8)
C35—O18—Na3	110.6 (5)	O17—C32—H32A	109.7
C37—S1—Na1	132.6 (4)	C31—C32—H32A	109.7
C38—S2—Na2	92.4 (3)	O17—C32—H32B	109.7
C39—S3—Na3	120.7 (3)	C31—C32—H32B	109.7
O1—C1—C2	115.0 (14)	H32A—C32—H32B	108.2
O1—C1—H1A	108.5	O17—C33—C34	109.0 (8)
C2—C1—H1A	108.5	O17—C33—H33A	109.9
O1—C1—H1B	108.5	C34—C33—H33A	109.9
C2—C1—H1B	108.5	O17—C33—H33B	109.9
H1A—C1—H1B	107.5	C34—C33—H33B	109.9
O2—C2—C1	110.2 (13)	H33A—C33—H33B	108.3
O2—C2—H2A	109.6	O18—C34—C33	110.7 (8)
C1—C2—H2A	109.6	O18—C34—H34A	109.5
O2—C2—H2B	109.6	C33—C34—H34A	109.5
C1—C2—H2B	109.6	O18—C34—H34B	109.5
H2A—C2—H2B	108.1	C33—C34—H34B	109.5
O2—C3—C4	114.9 (13)	H34A—C34—H34B	108.1
O2—C3—H3A	108.6	O18—C35—C36	109.6 (8)
C4—C3—H3A	108.6	O18—C35—H35A	109.8
O2—C3—H3B	108.5	C36—C35—H35A	109.7
C4—C3—H3B	108.5	O18—C35—H35B	109.7
H3A—C3—H3B	107.5	C36—C35—H35B	109.8
O3—C4—C3	110.8 (13)	H35A—C35—H35B	108.2
O3—C4—H4A	109.5	O13—C36—C35	110.2 (8)
C3—C4—H4A	109.5	O13—C36—H36A	109.6
O3—C4—H4B	109.5	C35—C36—H36A	109.6
C3—C4—H4B	109.5	O13—C36—H36B	109.6
H4A—C4—H4B	108.1	C35—C36—H36B	109.6
O3—C5—C6	112.4 (14)	H36A—C36—H36B	108.1
O3—C5—H5A	109.1	N1—C37—S1	177.4 (9)
C6—C5—H5A	109.1	N2—C38—S2	178.4 (9)
O3—C5—H5B	109.1	N3—C39—S3	179.8 (9)
C6—C5—H5B	109.1	N4—C40—S4	179.7 (9)
H5A—C5—H5B	107.9	N5—C41—S5	178.7 (9)
O4—C6—C5	116.6 (14)	N6—C42—S6	179.3 (10)
O4—C6—H6A	108.1	C44—C43—Cl1	117.5 (9)
C5—C6—H6A	108.1	C44—C43—H43A	107.9
O4—C6—H6B	108.1	Cl1—C43—H43A	107.9
C5—C6—H6B	108.1	C44—C43—H43B	107.9
H6A—C6—H6B	107.3	Cl1—C43—H43B	107.9
O4—C7—C8	112.0 (13)	H43A—C43—H43B	107.2
O4—C7—H7A	109.2	C43—C44—Cl2	116.9 (10)
C8—C7—H7A	109.2	C43—C44—H44A	108.1
O4—C7—H7B	109.2	Cl2—C44—H44A	108.1
C8—C7—H7B	109.2	C43—C44—H44B	108.1
H7A—C7—H7B	107.9	Cl2—C44—H44B	108.1
O5—C8—C7	113.2 (13)	H44A—C44—H44B	107.3
O5—C8—H8A	108.9	Cl3—C45—C45^i^	92.6 (19)
C7—C8—H8A	108.9	Cl3—C45—H45A	113.2
O5—C8—H8B	108.9	C45^i^—C45—H45A	113.2
C7—C8—H8B	108.9	Cl3—C45—H45B	113.2
H8A—C8—H8B	107.7	C45^i^—C45—H45B	113.2
O5—C9—C10	113.1 (13)	H45A—C45—H45B	110.5
O5—C9—H9A	109.0		

Symmetry codes: (i) −*x*+2, −*y*, −*z*+1.
